# SUBJECTIVE COGNITIVE DECLINE AND DAILY AFFECTIVE WELL-BEING

**DOI:** 10.1093/geroni/igae098.3325

**Published:** 2024-12-31

**Authors:** Mijin Jeong, Jacqueline Mogle, Heejung Jang, Nikki Hill, Mindy Katz

**Affiliations:** Keimyung University, Daegu, Republic of Korea; Clemson University, Clemson, South Carolina, United States; Clemson University, Clemson, South Carolina, United States; The Pennsylvania State University, University Park, Pennsylvania, United States; Albert Einstein College of Medicine, Bronx, New York, United States

## Abstract

Subjective cognitive decline (SCD) is self-reported cognitive decline in the absence of objective cognitive deficits. Although SCD (especially SCD accompanied by worry about the decline) is considered as the earliest progress of dementia, less is known about daily experiences of affect among older adults with SCD. Considering depression is deeply associated with progress of dementia, it is important to understand how individuals with SCD have experienced daily affect. We examined differences in levels of daily affective well-being for older adults with and without SCD. In the Einstein Aging Study (EAS), a total of 210 participants reported whether they perceived SCD and if so, indicated whether were worried by this decline. Daily positive affect (PA) and daily negative affect (NA) were measured using 8 items (happy; relaxed; energetic, enjoy; anxiety; loneliness; depression; and frustration) each day for 14 continuous days (between May 2017 and March 2020). We tested hypotheses by using multilevel models (MLM). For daily PA, SCD (either with or without worry) was associated with lower average daily reports of total PA specifically driven by differences on the items “happy” and “relaxed” compared to individuals without SCD. For daily NA, endorsement of SCD with worry was associated with higher total NA, specifically for the items “anxiety,” “depression,” and “frustration” compared to individuals without SCD and those with SCD but without worry. This study helps to better understand the impact of perceiving early cognitive changes on different aspects of daily affect to identify targets for promoting psychological well-being among older adults.

